# Cancer survival for Aboriginal and Torres Strait Islander Australians: a national study of survival rates and excess mortality

**DOI:** 10.1186/1478-7954-12-1

**Published:** 2014-01-31

**Authors:** John R Condon, Xiaohua Zhang, Peter Baade, Kalinda Griffiths, Joan Cunningham, David M Roder, Michael Coory, Paul L Jelfs, Tim Threlfall

**Affiliations:** 1 Menzies School of Health Research, Charles Darwin University, Darwin, NT, Australia; 2 Northern Territory Department of Health, Darwin, NT, Australia; 3 Cancer Council Queensland, Brisbane, QLD, Australia; 4 University of Sydney, Sydney, NSW, Australia; 5 University of South Australia, Adelaide, SA, Australia; 6 Murdoch Children’s Research Institute, University of Melbourne, Melbourne, Vic, Australia; 7 Australian Bureau of Statistics, Canberra, ACT, Australia; 8 Department of Health Western Australia, Western Australian Cancer Registry, Perth, WA, Australia

**Keywords:** Cancer, Survival, Australia, Australian Aboriginal, Torres Strait Islander, Indigenous Australian, Relative survival, Cause-specific survival

## Abstract

**Background:**

National cancer survival statistics are available for the total Australian population but not Indigenous Australians, although their cancer mortality rates are known to be higher than those of other Australians. We aimed to validate analysis methods and report cancer survival rates for Indigenous Australians as the basis for regular national reporting.

**Methods:**

We used national cancer registrations data to calculate all-cancer and site-specific relative survival for Indigenous Australians (compared with non-Indigenous Australians) diagnosed in 2001-2005. Because of limited availability of Indigenous life tables, we validated and used cause-specific survival (rather than relative survival) for proportional hazards regression to analyze time trends and regional variation in all-cancer survival between 1991 and 2005.

**Results:**

Survival was lower for Indigenous than non-Indigenous Australians for all cancers combined and for many cancer sites. The excess mortality of Indigenous people with cancer was restricted to the first three years after diagnosis, and greatest in the first year. Survival was lower for rural and remote than urban residents; this disparity was much greater for Indigenous people. Survival improved between 1991 and 2005 for non-Indigenous people (mortality decreased by 28%), but to a much lesser extent for Indigenous people (11%) and only for those in remote areas; cancer survival did not improve for urban Indigenous residents.

**Conclusions:**

Cancer survival is lower for Indigenous than other Australians, for all cancers combined and many individual cancer sites, although more accurate recording of Indigenous status by cancer registers is required before the extent of this disadvantage can be known with certainty. Cancer care for Indigenous Australians needs to be considerably improved; cancer diagnosis, treatment, and support services need to be redesigned specifically to be accessible and acceptable to Indigenous people.

## Introduction

Reports from several Australian states and territories indicate that cancer mortality rates are higher for Aboriginal and Torres Strait Islander peoples (hereafter respectfully referred to as “Indigenous Australians”) than other Australians for all cancers combined and for several cancer sites [1,2]. These elevations in Indigenous mortality rates are partly due to higher incidence rates for some (but not all) cancer sites [3] and partly to lower survival rates for many cancer sites [4-6]. A recent study in Queensland reported that five-year cancer survival (all sites combined) was considerably lower for Indigenous than non-Indigenous people (50.3% compared to 61.9%) [6].

Until recently no reliable national cancer incidence or survival statistics have been available for Indigenous Australians. As part of a project auspiced by the Australasian Association of Cancer Registries to establish national reporting of cancer statistics for Indigenous Australians, we recently reported the first assessment of completeness of Indigenous identification in national cancer registrations data and published seminational cancer incidence rates (covering 84% of the Indigenous population); this study confirmed that higher incidence rates are partly responsible for higher mortality rates for lung and other smoking-related cancers, and cervix, uterus, and liver cancers [3]. We now report national figures for cancer survival for Indigenous Australians.

Population-based cancer survival statistics are usually calculated using the relative survival method [7]. Relative survival analysis requires detailed life tables for the general population to calculate background probability of death. Relative survival analysis for Indigenous people with cancer has major limitations because life tables for the Indigenous population are only available for the single period 2005-2007 and not available by remoteness category. Previously published life tables were calculated using an inappropriate method that produced inconsistent results [8].

We therefore used relative survival analysis to calculate national cancer survival for Indigenous Australians for the period 2001-2005 using the 2005-2007 life tables and used cause-specific survival analysis to examine time trends over 15 years and compare cancer survival in urban with rural and remote areas. Relative survival analysis is the standard method to calculate population-based survival rates but is not without its limitations; cause-specific survival analysis is also appropriate in the right circumstances, particularly if reliable cause of death data are available [9]. We validated cause-specific analysis by comparison with relative survival analysis in a restricted analysis using the limited Indigenous life tables that are available.

This study and the previously reported seminational cancer incidence statistics [3] provide a template for regular national reporting of cancer statistics for Indigenous Australians.

## Methods

### Data

Cancer registrations data for all Australians diagnosed with cancer between 1 January 1991 and 31 December 2005 was obtained from the National Cancer Statistics Clearing House for the following data items: sex; date of birth; Indigenous status; remoteness of residence category, classified in the five categories of the Accessibility/Remoteness Index of Australia (ARIA) [10]; date of diagnosis; cancer site, coded according to the International Classification of Diseases Version 10 (ICD-10) [11]; date of death; and underlying cause of death (as recorded by cancer registries). Information on stage at diagnosis was not available for most cancer sites.

The exclusion criteria were the same as used in the most recent national report of cancer survival statistics for Australia; cancers diagnosed at the time of death were excluded and people diagnosed with multiple primary cancers were included as multiple records [7]. “Cancer site” refers to either the location within the body of the primary tumor, or, for cancers that do not originate at a particular location (such as cancers of the blood and lymphatic systems) the morphological type of the cancer. Cancer site was categorized at the ICD-10 three-digit level with some sites including multiple related three-digit categories (e.g., colorectal cancer: C18, C19 and C20) consistent with those used in the national survival report. Vital status was verified by matching the national cancer registrations dataset to the National Death Index for deaths occurring up to 31 December 2007. To reduce the impact of unreliable survival curves for longer follow-up intervals due to small numbers, we truncated survival times at five years after diagnosis.

Indigenous status is not included in pathology request/report forms (the primary source of notifications to cancer registries), so cancer registers obtain Indigenous status data from other notification sources (hospitals, radiation oncology centers, death notifications, etc). The completeness of Indigenous identification varies among the eight Australian state-based cancer registries; completeness is high for four registries since 1998 or earlier (New South Wales, Northern Territory, Queensland, and Western Australia) but less complete for the other four [3]. All registries were included in this study. All people identified as Indigenous were included in the “Indigenous” category; all others, including those with unknown or not stated Indigenous status, were included in the “non-Indigenous” category.

Data from one registry (Victoria) were excluded from cause-specific analyses because 77% of deceased cases did not have cause of death recorded; the other seven states and territories included 94% of the Indigenous Australian population in 2006 [12]. Cause of death was coded using the ICD-10 by five cancer registries. One registry coded cause of death using the International Classification of Diseases (Oncology) Version 3 (ICD-O-3) topography codes; [13] these were mapped and recoded to ICD-10 codes. One registry used ICD-10 codes for most deaths but used ICD-O-3 morphology codes for deaths caused by cancers of blood and lymphatic systems. Consequently, for all seven registries, all deaths from cancers of blood and lymphatic systems were grouped together and treated as a single cause of death.

### All-cancer and site-specific survival

Cancer survival (i.e., the proportion of cancer cases still alive) was estimated at one and five years after diagnosis. Relative survival was calculated for all cancers combined and for specific cancer sites, for cases diagnosed in 2001-2005, which were followed-up to the end of 2007. Non-Indigenous survival was adjusted to the age distribution of Indigenous cases: for all cancers combined, to the age-distribution of all Indigenous cases; for specific cancer sites, to the age-distribution of Indigenous cases for each site.

For non-Indigenous cases, background population probability of death by sex, age, and year was obtained from life tables for the total Australian population published by the Australian Bureau of Statistics (ABS) [14]. For Indigenous cases a life table for the period 2005-2007 was used because no consistent time series of life tables was available for earlier years [15]. Expected survival was estimated using the Ederer II method [16].

### Validation of cause-specific analysis

Relative survival analysis could not be used for some analyses because appropriate Indigenous life tables were not available, as described above. Therefore, cause-specific survival analysis was assessed to see whether it would give comparable results to relative survival analysis. The cause-specific survival rate and the relative survival rate estimate the same measure: the proportion of people that have not died from their cancer at the specified time after diagnosis. Cause-specific survival analysis uses only deaths for which the underlying cause of death is the same as the diagnosed cancer as the endpoint; relative survival analysis compares the crude survival rate with the background survival rate for the general population (adjusted to the age-sex distribution of the cohort of cancer cases).

For cause-specific analysis, a broad definition of cause-specific death was applied that included death from: cancer in the same region of the body as that of the primary site; cancer of unknown primary cancer site; or unknown cause. Of deaths counted as cause-specific under this definition: 88.9% were deaths for which the diagnosis site and cause of death agreed at ICD-10 three-digit level or both the diagnosis site and cause of death were cancers of the blood and lymphatic system; 0.9% were deaths from other cancers in the same regional grouping as the diagnosis site; 8.8% were deaths from cancer of unknown primary site; and 1.4% were deaths from unknown causes.

Cause-specific was compared with relative survival analysis for the period 2001-2005, for Australia excluding Victoria, for all cancers combined, and for specific cancer sites, separately for Indigenous and non-Indigenous cases. The survival rates and regression analysis results produced by the two methods were compared for: the all-cancer survival rate by year after diagnosis (not adjusted for site or age); the five-year survival rate for specific cancer sites (not adjusted for age); and the hazard ratios from proportional hazards regression (for cause-specific survival) and Poisson regression (for relative survival [17]) models. Regression models included terms for: Indigenous status; sex; age at diagnosis; cancer site; year since diagnosis (as indicator terms); and interaction terms for Indigenous status with each of age at diagnosis and year since diagnosis. Cause-specific analysis was found to be comparable to relative survival analysis for all cancers combined, but not for some specific cancer sites (see results). Therefore time trends and regional variation in cancer survival were investigated using proportional hazard regression of cause-specific death rates for all cancers combined and for the four most prevalent cancer sites in the Indigenous population (excluding head and neck cancer, for which cause-specific and relative survival did not produce similar results).

### Regression analysis

Cox proportional hazards regression was used to analyze cancer mortality of cases diagnosed in 1991-2005 for Australia, excluding Victoria. The model included terms for: Indigenous status, sex, age at diagnosis, and ARIA remoteness category (the four terms of a priori interest); cancer site; and interaction terms for Indigenous status with each of age at diagnosis (base age 59 years) and remoteness categories (base category “major cities”). These interaction terms were included because the effects of age at diagnosis and remoteness of residence (but not sex) were found to be different for Indigenous compared with non-Indigenous people. Age at diagnosis and ARIA remoteness category were each included as a single ordinal term; including them as categorical variables did not improve model fit. Cancer site was included as multiple indicator terms. Scaled Schoenfeld residuals, which test for nonzero slope over time, were used to check if the proportional hazards assumptions of each variable were satisfied. A step function of Indigenous status with follow-up time (as annual intervals) was also included in the model because the proportional hazards assumption was not met for Indigenous status, as has been demonstrated previously [6].

### Time trends

For time trends, follow-up was limited to the first two years after diagnosis because all subjects had at least two years of potential follow-up (so that shorter follow-up time for subjects diagnosed late in the study period did not bias time trends) and because most of the excess mortality of Indigenous cases occurred in the first two years after diagnosis (see results). Time trends were analyzed for all cancers combined (adjusted for cancer site) and for four of the most prevalent cancer sites in the Indigenous population: colorectal, lung, breast (female only), and prostate. Two-year cause-specific survival for all cancers combined (adjusted for age and cancer site) was calculated by Indigenous status, year of diagnosis, and ARIA category. Multivariate analysis was performed using proportional hazards regression. The model included terms for: Indigenous status, sex, age at diagnosis, and year of diagnosis (the terms of *a priori* interest); cancer site (for analysis of all cancers combined); and interaction terms for Indigenous status with each of age at diagnosis and year of diagnosis. Variation in time trend by remoteness category was investigated by including an interaction term for ARIA category by year in separate models for Indigenous and non-Indigenous cases.

All analyses were performed using Stata (versions 11.1 and 12.1) [18]. Relative survival analysis was performed using the ‘strs’ procedures of Stata [17]. Ethics approvals were obtained from 12 human research ethics committees covering all states and territories, including several Indigenous committees or subcommittees. Approval to use cancer registrations data was obtained from each of the eight Australian cancer registries.

## Results

1,235,592 Australians diagnosed with invasive cancer between 1991 and 2005 met the inclusion criteria for this study; 0.6% were identified as Indigenous (Aboriginal and/or Torres Strait Islander peoples) (Table 1). Indigenous cases were more likely to be female, younger, and live outside major cities. A higher proportion of Indigenous (67%) than non-Indigenous (55%) cases died by the end of 2007.

**Table 1:** Demographic characteristics of people diagnosed with cancer, Australia, 1991-2005

	**Indigenous**	**Non-indigenous**
	**n = 7,019**	**n = 1,228,573**
	***Percent of column total***	
*Sex*		
Male	47.8	55.0
Female	52.2	45.0
*Age at diagnosis*		
0 to 49 years	28.9	15.1
50 to 59 years	23.1	15.7
60 to 69 years	24.4	24.0
70 years and over	23.6	45.2
Median age (years)	59	68
*State of residence*		
Queensland	30.7	18.8
New South Wales	27.8	34.4
Western Australia	16.2	8.9
Northern Territory	15.5	0.4
Victoria	4.8	24.7
South Australia	4.1	8.8
Tasmania	0.6	2.7
Australian Capital Territory	0.3	1.3
*Remoteness of residence*		
Major cities	27.3	65.9
Inner regional	15.8	22.5
Outer regional	25.3	9.8
Remote	11.2	1.1
Very remote	19.8	0.4
Other^1^	0.7	0.4
*Vital status at 31*/*12*/*2007*		
Alive	33.4	45.1
Dead	66.6	54.9

^1^Includes migratory and unknown.

### Survival rates

For all cancers combined (adjusted for age), survival was lower for Indigenous than other Australians (Table 2). Most of the difference arose during the first year after diagnosis; survival was 63.8% for Indigenous compared with 83.4% for non-Indigenous cases at one year after diagnosis and 46.7% compared with 70.0% at five years. Survival was lower for Indigenous than non-Indigenous cases for almost all cancer sites at both one and five years after diagnosis.

**Table 2:** **One**-**year and five**-**year relative survival**^**1 **^**by Indigenous status and cancer site**/**type**, **Australia**, **2001**-**2005**

	**Indigenous**					**Non-indigenous**^**2**^				
	**Cases**	**One-year**		**Five-year**		**Cases**	**One-year**		**Five-year**	
**Cancer site/type**	**n**	**%**	**(95% CI)**	**%**	**(95% CI)**	**n**	**%**	**(95% CI)**	**%**	**(95% CI)**
Head and neck (C1-14, C30-32)	236	60.3	(53.6-66.4)	32.4	(25.6-39.5)	11 893	86.1	(85.5-86.8)	65.9	(64.9-66.9)
Stomach (C16)	73	39.8	(28.3-51.1)	16.9	(8.0-29.0)	9 349	56.1	(55.1-57.1)	30.7	(29.6-31.7)
Colorectal (C18-20)	306	80.1	(74.8-84.6)	58.5	(50.9-65.7)	62 021	85.6	(85.3-85.9)	66.3	(65.9-66.8)
Anus (C21)	22	73.8	(49.9-88.0)	49.1	(20.9-73.8)	1 304	91.7	(89.9-93.1)	70.4	(67.3-73.4)
Liver (C22)	83	24.8	(16.0-34.6)	11.2	(5.1-20.0)	4 500	43.4	(41.9-44.9)	19.9	(18.5-21.2)
Pancreas (C25)	91	26.3	(17.6-35.9)	11.2	(5.4-19.5)	9 667	27.6	(26.7-28.5)	7.6	(7.0-8.3)
Lung (C33-34)	500	29.4	(25.4-33.5)	9.1	(6.3-12.5)	42 139	43.4	(42.9-43.9)	16.7	(16.3-17.2)
Melanoma (C43)	59	95.6	(84.4-100.7)	73.8	(55.5-88.5)	48 592	97.9	(97.7-98.1)	92.4	(91.9-92.8)
Breast (C50)	420	94.1	(91.0-96.4)	80.1	(74.3-85.2)	59 640	97.9	(97.7-98.0)	89.5	(89.1-89.8)
Cervix (C53)	109	79.8	(70.8-86.5)	57.6	(46.6-67.4)	3 475	90.8	(89.7-91.7)	78.3	(76.7-79.8)
Uterus (C54)	96	85.2	(75.9-91.4)	78.7	(66.7-88.2)	7 969	94.0	(93.5-94.6)	84.6	(83.6-85.7)
Ovary (C56)	53	77.1	(62.9-86.8)	50.1	(33.6-65.1)	5 777	83.5	(82.5-84.5)	53.1	(51.6-54.6)
Prostate (C61)	181	90.4	(84.0-94.9)	83.6	(72.5-93.2)	68 475	97.3	(97.1-97.4)	91.0	(90.6-91.4)
Testis (C62)	25	100.5	(100.5-100.5)	94.5	(73.6-100.5)	3 209	98.8	(98.3-99.2)	96.9	(96.0-97.6)
Kidney (C64)	69	77.5	(65.2-86.3)	68.1	(53.0-80.7)	10 522	84.9	(84.2-85.6)	73.2	(72.1-74.2)
Bladder (C67)	53	61.5	(46.4-74.0)	54.7	(36.1-72.6)	11 270	85.2	(84.5-85.9)	68.1	(66.9-69.2)
Brain (C71)	48	58.9	(43.6-71.4)	42.2	(26.9-56.9)	6 800	64.3	(63.2-65.5)	40.4	(39.2-41.7)
Thyroid (C73)	74	93.0	(83.9-97.5)	85.0	(69.3-94.5)	6 809	97.0	(96.5-97.4)	96.0	(95.3-96.6)
Hodgkin lymphoma (C81)	26	96.8	(76.2-100.1)	100.6	(79.2-104.0)	2 290	96.0	(95.1-96.8)	90.3	(88.8-91.7)
NHL (C82-85, C96)	101	67.4	(56.9-76.1)	52.0	(37.3-65.8)	18 392	85.5	(85.0-86.0)	73.7	(72.9-74.5)
Leukemia (C91-95)	108	64.6	(54.5-73.1)	53.7	(42.7-63.9)	12 864	80.3	(79.5-81.0)	63.9	(62.9-64.9)
Unknown primary (C76-80)	196	29.2	(22.8-35.8)	14.9	(9.9-21.1)	14 997	35.6	(34.9-36.4)	21.3	(20.6-22.1)
Other	363	61.1	(55.7-66.1)	40.3	(34.4-46.2)	42 463	78.5	(78.1-78.9)	57.2	(56.7-57.8)
All cancers (C00-96, D45-47)	3 292	63.8	(62.1-65.5)	46.7	(44.6-48.7)	464 417	83.4	(83.3-83.5)	70.0	(69.8-70.2)

^1^Relative survival rate.

^2^Age-adjusted to age distribution of Indigenous cases for each cancer site.

### Cause-specific compared with relative survival

**Figure 1:**
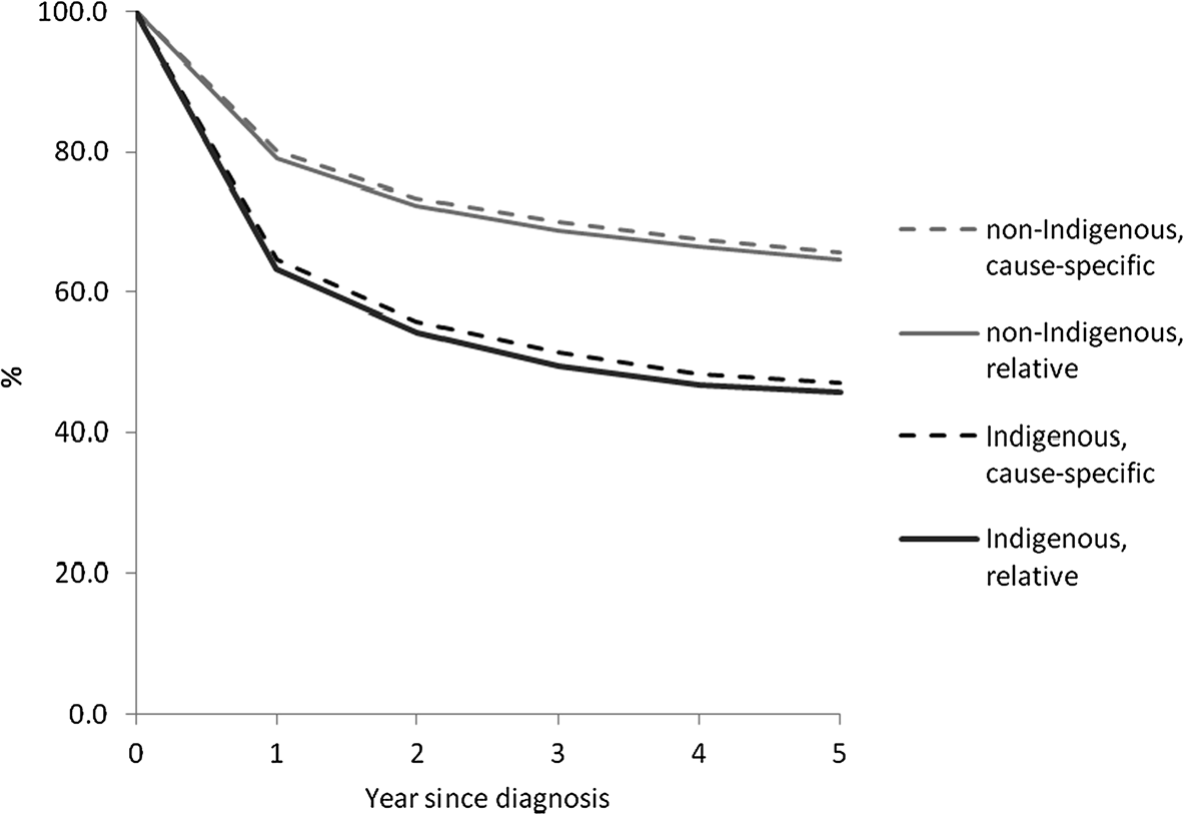
Cause-specific compared with relative survival, all cancers combined by Indigenous status, Australia (excluding Victoria), 2001-2005.

For all cancers combined, cause-specific survival was similar to relative survival for both Indigenous and non-Indigenous cases throughout the first five years after diagnosis, with cause-specific survival slightly higher than relative survival (Figure 1). For non-Indigenous cases cause-specific and relative survival were 80.1% (95% CI 80.0-80.3) compared with 79.1% (78.9-79.2) at one year and 65.6% (65.4-65.8) compared with 64.6% (64.4-64.8) at five years, while for Indigenous cases 64.7% (63.0-66.4) compared with 63.2% (61.4-64.9) at one year and 47.0% (45.0-48.9) compared with 45.8% (43.7-47.9) at five years. Results of cause-specific analysis using proportional hazards regression were similar to those of relative survival analysis using Poisson regression (Table 3, Additional file 1: Table S3a); hazard ratios were similar for all terms included in the models (with the same terms in each model). For specific cancer sites, the cause-specific survival rate was similar to the relative survival rate for most sites but very different for some; differences were greater for Indigenous than non-Indigenous cases (see Additional file 2: Table S7). Cause-specific analysis was therefore restricted to analysis of all cancers combined and selected cancer sites.

**Table 3:** **Cause**-**specific compared with relative survival regression analysis**^**1**^, **all cancers combined**, **Australia (excluding Victoria)**, **2001**-**2005**

	**Relative**		**Cause-specific**	
	**HR**^**2**^	**(95% CI)**	**HR**	**(95% CI)**
Indigenous				
1st year after diagnosis	1.88	(1.77-2.00)	1.94	(1.82-2.06)
2nd year after diagnosis	1.63	(1.43-1.85)	1.64	(1.45-1.86)
3rd year after diagnosis	1.66	(1.35-2.03)	1.62	(1.35-1.95)
4th year after diagnosis	1.42	(1.02-1.96)	1.66	(1.28-2.15)
5th year after diagnosis	0.65	(0.30-1.38)	0.96	(0.60-1.52)
Sex				
Female	0.92	(0.91-0.93)	0.92	(0.91-0.94)
Age at diagnosis (per year of age)				
Non-indigenous	1.03	(1.03-1.03)	1.03	(1.03-1.03)
Indigenous	1.02	(1.01-1.02)	1.02	(1.01-1.02)

^1^Both models were also adjusted for cancer site (see Additional file 1: Table S3a).

^2^Hazard ratio.

### Regression analysis

For all cancers combined, the cause-specific death rate was higher (i.e., cancer survival was lower) for males than females; for older than younger cases; and for remote than urban residents (Table 4, Additional file 3: Table S4a). The effects of age at diagnosis and remoteness were different for Indigenous than non-Indigenous cases. The death rate increased by 3% per year of age for non-Indigenous cases but by 2% for Indigenous cases; this was because death rates were relatively high for younger Indigenous cases. Death rates were higher for those resident in very remote compared with metropolitan areas: 23% higher for non-Indigenous but 65% higher for Indigenous cases. The effect of sex was similar in Indigenous and non-Indigenous cases; the hazard ratio for an interaction term for Indigenous status by sex was not statistically significant, so an interaction term for “Indigenous status by sex” was not included in the final model.

**Table 4:** **Regression analysis**^**1 **^**of cause**-**specific mortality for all cancers combined**, **Australia (excluding Victoria)**, **1991**-**2005**

	**HR**^**2**^	**(95%CI)**
Indigenous^3^		
1st year after diagnosis	1.50	(1.41-1.60)
2nd year after diagnosis	1.23	(1.11-1.35)
3rd year after diagnosis	1.21	(1.07-1.38)
4th year after diagnosis	1.16	(0.98-1.37)
5th year after diagnosis	0.99	(0.79-1.23)
Sex		
Female	0.93	(0.92-0.93)
Age at diagnosis (per year of age)		
Non-indigenous	1.03	(1.03-1.03)
Indigenous	1.02	(1.02-1.02)
Remoteness (per ARIA category)		
Non-indigenous	1.05	(1.05-1.06)
Indigenous	1.13	(1.11-1.16)

^1^The model was also adjusted for cancer site (see Additional file 3: Table S4a).

^2^Hazard ratio.

^3^Applies to the reference categories of the interaction terms (i.e., people of median age 59 years and resident in major cities).

### Time trend in two-year survival rate

Cancer survival improved considerably for non-Indigenous cases, but less so for Indigenous cases. The death rate in the first two years after diagnosis decreased by 2% per year for non-Indigenous (28% decrease over 15 years), but by only 1% per year for Indigenous cases (11% over 15 years) (Table 5, Additional file 4: Table S5a).

**Table 5:** **Time trends**: **regression analysis**^**1 **^**of cause**-**specific mortality in two years after diagnosis for all cancers combined**, **Australia** (**excluding Victoria**), **1991**-**2005**

	**HR**^**2**^	**(95% CI)**
Indigenous^3^	1.54	(1.42-1.66)
Sex		
Female	0.95	(0.94-0.95)
Age at diagnosis (per year of age)		
Non-indigenous	1.03	(1.03-1.03)
Indigenous	1.02	(1.02-1.02)
Remoteness (per ARIA category)		
Non-indigenous	1.06	(1.06-1.07)
Indigenous	1.16	(1.13-1.19)
Year of diagnosis (per year)		
Non-indigenous	0.98	(0.98-0.98)
Indigenous	0.99	(0.98-1.00)

^1^The model was also adjusted for cancer site (see Additional file 4: Table S5a).

^2^Hazard ratio.

^3^Applies to the reference categories of the interaction terms (i.e., people of median age 59 years in 2005).

**Figure 2:**
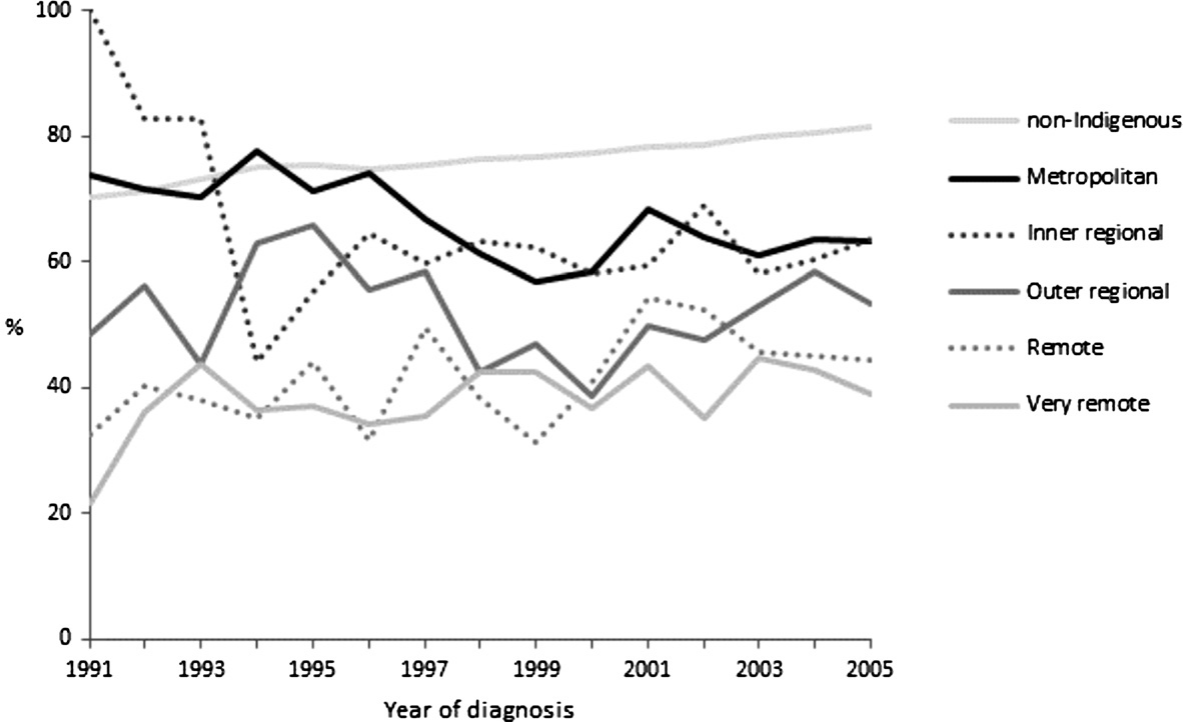
Two-year cause-specific survival rate by year of diagnosis, all cancers combined, indigenous by ARIA category and non-indigenous, Australia (excluding Victoria), 1991-2005.

For non-Indigenous cases this decrease over time was similar for all ARIA categories (hazard ratio [HR] for interaction term “year by ARIA category”: 1.00, 95% CI 1.00-1.00) (see Additional file 5: Table S8). For Indigenous cases survival increased for residents of remote and very remote areas but not for urban residents (Figure 2). This was confirmed by multivariate analysis (see Additional file 5: Table S8); the time trend was different for urban compared with rural and remote residents (HR for interaction term “year by ARIA category”: 0.992, 95% CI 0.986-0.998). The death rate decreased over time for Indigenous cases residing in more remote areas, by 26% between 1991 and 2005 in the “very remote” ARIA category (HR 0.98 per year, 95% CI 0.97-0.99), but did not decrease over time for Indigenous cases resident in the “major metropolitan” ARIA category (HR 1.01, 95% CI 0.99-1.03).

Time trends were also examined for four of the most prevalent cancers in the Indigenous population: colorectal, lung, breast (female only), and prostate (Table 6). For non-Indigenous cases death rates decreased for all four cancers. For Indigenous people with colorectal and breast cancer, death rates decreased to a similar extent as for non-Indigenous cases, but there was no decrease for lung and prostate cancer.

**Table 6:** **Time trends**: **regression analysis of cause**-**specific mortality in two years after diagnosis for specific cancers**, **hazard ratio** (**95% confidence interval**), **Australia** (**excluding Victoria**), **1991**-**2005**

	**Colorectal**		**Lung**		**Breast**		**Prostate**	
Indigenous^1^	1.44	(1.05-1.97)	1.42	(1.21-1.66)	1.66	(1.06-2.61)	5.59	(3.15-9.94)
Sex								
Female	1.02	(1.00-1.04)	0.90	(0.89-0.92)	n/a		n/a	
Age at diagnosis								
Non-indigenous	1.03	(1.02-1.03)	1.02	(1.02-1.02)	1.04	(1.04-1.04)	1.08	(1.08-1.08)
Indigenous	1.00	(0.99-1.01)	1.01	(1.01-1.02)	1.00	(0.99-1.02)	1.02	(1.00-1.05)
Remoteness (per ARIA category)								
Non-indigenous	1.08	(1.06-1.09)	1.07	(1.06-1.08)	1.06	(1.03-1.10)	1.13	(1.10-1.16)
Indigenous	1.19	(1.07-1.33)	1.08	(1.03-1.14)	1.29	(1.13-1.48)	1.35	(1.12-1.64)
Year of diagnosis (per year)								
Non-indigenous	0.97	(0.97-0.98)	0.99	(0.99-0.99)	0.96	(0.95-0.96)	0.96	(0.95-0.96)
Indigenous	0.97	(0.94-1.00)	1.00	(0.98-1.02)	0.97	(0.92-1.02)	1.06	(0.99-1.13)

^1^Applies to the reference categories of the interaction terms (i.e., people of median age 59 years in 2005).

## Discussion

Survival is lower for Indigenous than other Australians with cancer, for all sites combined, and for many individual cancer sites. This disparity is greatest immediately after diagnosis, greater for remote than metropolitan residents, greater for younger than older people, and is increasing over time.

### Cause-specific survival analysis

Although not without potential limitations, [9] relative survival is the preferred method for population-based cancer survival analysis (as distinct from clinical studies) because of concerns about accuracy of classification of cause of death, and because for many cancer cases, cancer is not the only condition contributing to their death. However, the inadequacies of life tables for the Australian Indigenous population limit the applicability of the relative survival method for analysis of time-trends and variation by remoteness of residence. The ABS has determined that the non-standard methods used to calculate Indigenous life tables before 2005 [19] were unreliable and produced considerably inaccurate results [20]. The ABS has subsequently published Indigenous life tables for 2005-2007 using the standard method used for the total Australian population but has not published a retrospective time series [15] or published Indigenous life tables stratified by remoteness category (but is expected to do so in late 2013).

Life tables stratified by Indigenous status also have a potential for differential misclassification of Indigenous status between the life tables and the cancer registration data. If, for example, Indigenous status was less complete in the deaths data used to calculated probability of death in life tables than in cancer registers, the life tables would underestimate probability of death for the background Indigenous population, and consequently, relative survival analysis would underestimate cancer survival. Death and cancer registers both rely on death notifications and hospital records (for deaths occurring in hospital) for Indigenous status data, so this potential differential misclassification may not be large, but there is no evidence available to test this.

Over time, improvements in the accuracy of Indigenous mortality data may provide more detailed and consistent Indigenous life tables that will enable use of relative survival for more detailed analysis of survival for Indigenous people with cancer. In the interim, our comparison of cause-specific with relative survival analysis indicates that cause-specific analysis is as reliable as relative survival for analysis of time trends and regional variation in cancer survival for all cancers combined and some specific cancer sites.

For all cancers combined and for most cancer sites relative survival and cause-specific survival produced similar results (see Additional file 2: Table S7 and Additional file 5: Table S8), but for some specific sites such as head and neck, bladder, and leukemia the two methods produced very different results. Where there was a large difference between the two methods, cause-specific survival was mostly higher than relative survival. Underestimation of cancer-related deaths for people with these cancers would produce this effect, either because deaths due to cancer were misclassified or a high proportion of deaths were partially attributable to these cancers but few were classified as cancer deaths [9]. Alternatively, for these sites relative survival might be underestimated because the probability of noncancer related death for people with these cancers is higher than for the general population. This is plausible for head and neck cancer and bladder cancer because they are smoking-related, and the probability of noncancer death for people with these cancers would be higher than that of the general population (most of whom are not smokers). However, this is not consistent with the results for other smoking-related cancers such as lung and pancreas, for which relative and cause-specific survivals were similar. It is not obvious why relative and cause-specific survivals are so different for some cancer sites.

### Limitations

Identification of Indigenous people in cancer registrations data is known to be high for four of the eight registries included in this study (New South Wales, Northern Territory, Queensland, and Western Australia), which cover 84% of the Indigenous population [3]. We have included data from the other four registries with low identification of Indigenous people (Australian Capital Territory, South Australia, Tasmania, and Victoria) (Table 1). Consequently it is likely that a proportion of Indigenous people were misclassified as non-Indigenous in this study. Indigenous cases comprised 0.6% of cases, which is less than the proportion of Indigenous people in the total Australian population (0.9% in the 50+ age-group in 2006), [12] but cancer incidence is different for many cancer sites for Indigenous than other Australians, including being lower for several of the most common cancers (breast, colon and rectum, prostate, and melanoma) [3] so a direct comparison of population proportions is not informative. We estimate that up to 0.2% of the non-Indigenous group may be misclassified Indigenous cases, indicating that up to 25% of Indigenous cases may be misclassified as non-Indigenous. If misclassified Indigenous cases had similar cancer survivals to other Indigenous cases, then the Indigenous survivals reported here are unbiased. If misclassified Indigenous cases had better survival than those correctly classified as Indigenous, perhaps because those incorrectly classified had better social, economic and environmental circumstances than those correctly classified as Indigenous, the Indigenous survivals reported here would be an underestimate to a small extent. Including misclassified Indigenous cases in the non-Indigenous group would have had an insignificant effect on non-Indigenous survival.

This study does not include people diagnosed with cancer after 2005, and follow-up of vital status after 2007, because national coded data on cause of death produced by the ABS has not been available to cancer registries since 2007 when data providers decided that the previous process to request and approve data access was no longer adequate. Efforts to develop a new approval process have been underway for several years but have not yet been successful [21]. Until cause of death data becomes available to cancer registers again, cause-specific cancer survival for Indigenous Australians cannot be updated for recent years.

One potentially large source of bias in this study is an information bias arising because Indigenous status in cancer registration data is partially derived from deaths data. The primary source of case ascertainment, pathology reports, does not include Indigenous status. Cancer registers rely on notifications from hospitals and death registrations as their main sources of Indigenous status data. If notifications from hospitals are significantly incomplete, people identified as Indigenous solely from death registrations will introduce differential misclassification of Indigenous status; Indigenous cases who have died will be more likely to be identified as Indigenous than Indigenous cases who have not died. The national cancer registration dataset does not include data on the source(s) of Indigenous status for each cancer registration, so the extent of this potential bias could not be assessed by this study; this requires specific investigation with cancer registries.

### Reasons for lower survival

This study confirms, at the national level, previous reports from individual states/territories that cancer survival is lower for Indigenous than other Australians [4,22,23]. These studies have found that Indigenous people are more likely to have advanced disease when diagnosed, more likely to have chronic disease comorbidity, and less likely to be offered, choose, and complete curative treatment. However, these factors only partly explained the lower survival of Indigenous people suggesting that other unmeasured factors were also involved. These might include the less advantageous social, educational, economic, and environmental circumstances of many Indigenous Australians [1].

Excess mortality of Indigenous (compared with non-Indigenous) cancer cases was greatest in the first year after diagnosis; by the fifth year Indigenous cases had no excess mortality. This confirms at the national level a similar finding reported from Queensland [6]. This may reflect delayed cancer diagnosis (with more Indigenous cases having disseminated disease when diagnosed), lower access to curative cancer treatment, or higher levels of chronic disease comorbidity that complicate or preclude cancer treatment; all these factors have been reported as more common for Indigenous than other Australians [2,23]. The information bias arising from Indigenous cases being differentially identified from death certificates (described above) might also partly explain this finding, although cases diagnosed at the time of death were excluded from this analysis so it is not obvious how this bias would operate more in the first year after diagnosis than in later years. This needs to be further investigated.

Remoteness of residence was associated with lower survival. For Indigenous cases this disparity was very large; death rates were 65% higher for Indigenous people in very remote areas than in major cities. Thirty-one percent of Indigenous cases live in remote or very remote areas, compared to only 1% of non-Indigenous cases, so remoteness is a particularly serious detrimental factor for Indigenous people with cancer. Survival increased for Indigenous cases in remote areas, but in 2005 was still lower than for urban Indigenous cases, for whom survival had not improved at all. Survival improved considerably for non-Indigenous cancer cases, with a 28% decrease in death rate between 1991 and 2005. Improvements in cancer diagnosis, treatment and support services that have been successful in improving cancer outcomes for most Australians in recent years have apparently been less effective for Indigenous people. Access to, and acceptability of, diagnosis and treatment services are likely to be part of the explanation for the geographic disparity; the previous studies cited above provide little information about this. Further investigation of the reasons for the very poor survival of cancer cases from rural and remote areas, and how to overcome them, should be a high priority for both Indigenous and non-Indigenous people.

Population screening can lead to overdiagnosis of cancers that would not have otherwise been diagnosed before the person died from other causes. This may account for some of the improvement in survival for non-Indigenous Australians for breast and prostate cancers and to a lesser extent colorectal cancer over the period of this study, during which screening for these cancers became more common (screening for colorectal cancer increased more recently). Indigenous Australians have lower cancer screening participation, [2,24] so they may be less susceptible to this overdiagnosis effect. There was little evidence of such an effect for breast and colorectal cancers in this study; the time trend in death rates was similar for Indigenous and non-Indigenous cases for both cancers (Table 6), but for prostate cancer the death rate decreased for non-Indigenous cases but not for Indigenous cases, which would be consistent with increasing overdiagnosis of prostate cancer among non-Indigenous cases only. For all cancers combined excluding these three screened cancers, the time trend for death rates (HR per year: non-Indigenous 0.97, Indigenous 0.98) was similar to that when the screened cancers were included (non-Indigenous 0.98, Indigenous 0.99).

In recent years there has been increased attention to the disadvantage suffered by Indigenous people with cancer. In 2010 Cancer Australia (the Australian Government’s cancer agency) commissioned a report on research priorities for Indigenous cancer control, [25] although it is still considering what action to take. A national roundtable on cancer control research for Indigenous Australians, convened in the same year by a senior Indigenous researcher, [26] led to the establishment of a national Centre of Research Excellence in Indigenous cancer control in 2012 [27]. The Centre has already established a National Indigenous Cancer Network of Indigenous cancer survivors and other community members, health professionals, and researchers [28]. Cancer services have started initiatives to improve cancer care in rural areas, such as the establishment of radiation oncology services in regional centres such as Darwin and Townsville [29] and use of telemedicine to provide access from remote communities to specialist oncologists [30]. Regular reporting of reliable Indigenous cancer survival statistics is essential to determine whether these initiatives are working.

## Conclusions

Cancer survival is lower for Indigenous than other Australians, for all cancers combined, and many individual cancer sites. This disparity is greatest in remote areas and has increased over time because survival for Indigenous people has not improved as much as for Australians generally. Cancer survival statistics for the total Australian population do not necessarily apply to Indigenous Australians who develop cancer; more specific information is needed to assist them in making important treatment and personal decisions and to assess the effectiveness of efforts to reduce this disparity. Cancer registration data can be used to produce regular national survival statistics for Indigenous Australians, as they are for Australians generally. Despite data limitations, reliable statistics on cancer survival for Indigenous Australians can and should be reported on a regular basis by national and state/territory cancer statistics agencies.

## Abbreviations

ABS: Australian bureau of statistics; ARIA: Accessibility/remoteness index of Australia; ICD-10: International classification of diseases, version 10; ICD-O-3: International classification of diseases for oncology, version 3.

## Competing interests

The authors declare that they have no competing interest.

## Authors’ contributions

JCo, JCu, DR, MC, PJ, and TT conceived and initiated the study. JCo, JCu, XZ, and PB refined the study design and undertook data management and/or analysis. KG assisted with data management and analysis. JCo, XZ, PB, and JCu helped draft the manuscript, with advice from DR, MC, PJ, KG, and TT. All authors read and approved the final manuscript.

## Supplementary Material

Additional file 1: Table S3a Cause-specific compared with relative survival regression analysis, all cancers combined, Australia (excluding Victoria) 2001-2005 (full model). Description: Table S3, including hazard ratios for specific cancer sites. Click here for file

Additional file 2: Table S7 Cause-specific compared with relative survival, five-year survival rate (%) by Indigenous status and site, Australia excluding Victoria, 2001-2005. Click here for file

Additional file 3: Table S4a Regression analysis of cause-specific mortality for all cancers combined, Australia (excluding Victoria) 1991-2005 (full model). Description: Table S4 including hazard ratios for specific cancer sites. Click here for file

Additional file 4: Table S5a Time trends: regression analysis of cause-specific mortality in two years after diagnosis for all cancers combined, Australia (excluding Victoria) 1991-2005 (full model). Description: Table S5 including hazard ratios for specific cancer sites. Click here for file

Additional file 5: Table S8 Regression analysis for all cancers combined including an interaction term for year of diagnosis by remoteness category, hazard ratio (95% confidence interval), Australia (excluding Victoria) 1991-2005. Description: Separate regression analyses for Indigenous and non-Indigenous cases including interaction term to assess whether time trends vary by remoteness of residence. Click here for file
