# Supplementary material for: Cancer survival for Aboriginal and Torres Strait Islander Australians: a national study of survival rates and excess mortality
Source: Popul Health Metr. 2014 Jan 31;12:1. doi: 10.1186/1478-7954-12-1 (PMC3909914; doi:10.1186/1478-7954-12-1)
Supplement: Additional file 1: Table S3a — Cause-specific compared with relative survival regression analysis, all cancers combined, Australia (excluding Victoria) 2001-2005 (full model). Description: Table S3, including hazard ratios for specific cancer sites. [file 1478-7954-12-1-S1.pdf]

Table 3a Cause-specific compared with relative survival regression analysis, all-cancers combined, Australia (excluding Victoria) 2001-2005 (full model).

	Cause-specific		Relative	
	**HR[1]**	**(95%CI)**	**HR**	**(95%CI)**
Indigenous				
1st year after diagnosis	1.88	(1.77-2.00)	1.94	(1.82-2.06)
2nd year after diagnosis	1.63	(1.43-1.85)	1.64	(1.45-1.86)
3rd year after diagnosis	1.66	(1.35-2.03)	1.62	(1.35-1.95)
4th year after diagnosis	1.42	(1.02-1.96)	1.66	(1.28-2.15)
5th year after diagnosis	0.65	(0.30-1.38)	0.96	(0.60-1.52)
Sex				
Female	0.92	(0.91-0.93)	0.92	(0.91-0.94)
Age at diagnosis (per year of age)				
Non-Indigenous	1.03	(1.03-1.03)	1.03	(1.03-1.03)
Indigenous	1.02	(1.01-1.02)	1.02	(1.01-1.02)
Cancer site/type[2]				
Head & neck	0.96	(0.92-1.00)	0.82	(0.79-0.86)
Stomach	3.27	(3.15-3.38)	3.06	(2.96-3.16)
Anus	1.10	(0.98-1.25)	1.04	(0.93-1.17)
Liver	5.60	(5.37-5.84)	4.95	(4.74-5.16)
Pancreas	7.66	(7.43-7.90)	6.90	(6.70-7.11)
Lung	4.88	(4.77-4.98)	4.47	(4.38-4.57)
Melanoma	0.19	(0.18-0.20)	0.24	(0.23-0.25)
Breast	0.28	(0.27-0.29)	0.33	(0.32-0.35)
Cervix	1.14	(1.05-1.23)	1.07	(0.98-1.15)
Uterus	0.49	(0.46-0.53)	0.53	(0.50-0.57)
Ovary	2.25	(2.15-2.36)	2.22	(2.13-2.33)
Prostate	0.16	(0.15-0.17)	0.26	(0.26-0.27)
Testis	0.19	(0.15-0.24)	0.18	(0.15-0.23)
Kidney	1.04	(0.99-1.09)	0.94	(0.90-0.98)
Bladder	0.93	(0.89-0.97)	0.80	(0.77-0.84)
Brain	6.41	(6.18-6.65)	5.99	(5.77-6.21)
Thyroid	0.18	(0.15-0.21)	0.19	(0.17-0.22)
Hodgkin lymphoma	0.72	(0.62-0.83)	0.72	(0.63-0.82)
Non-Hodgkin lymphoma	1.11	(1.07-1.15)	1.01	(0.97-1.04)
Leukaemia	1.81	(1.75-1.88)	1.26	(1.21-1.31)
Unknown primary	6.15	(5.99-6.32)	5.21	(5.08-5.35)
Others	1.99	(1.94-2.04)	1.62	(1.59-1.66)

1. Hazard ratio
2. compared to colorectal cancer.
