# Supplementary material for: Cancer survival for Aboriginal and Torres Strait Islander Australians: a national study of survival rates and excess mortality
Source: Popul Health Metr. 2014 Jan 31;12:1. doi: 10.1186/1478-7954-12-1 (PMC3909914; doi:10.1186/1478-7954-12-1)
Supplement: Additional file 2: Table S7 — Cause-specific compared with relative survival, five-year survival rate (%) by Indigenous status and site, Australia excluding Victoria, 2001-2005. [file 1478-7954-12-1-S2.pdf]

Table 7. Cause-specific compared with relative survival, five-year survival rate (%)[1] by Indigenous status and site, Australia excluding Victoria, 2001-2005.

	Indigenous			Non-Indigenous		
Cancer site/type	Relative	Cause-Specific	Difference[2]	Relative	Cause-Specific	Difference[2]
Head & neck	31.5	37.8	6.3	59.3	73.7	14.4
Stomach	15.0	15.9	0.9	27.7	27.9	0.2
Colorectal	58.0	52.9	-5.2	65.0	63.3	-1.7
Anus	47.7	59.2	11.6	64.3	65.9	1.6
Liver	10.2	12.7	2.5	14.4	16.6	2.2
Pancreas	10.5	9.7	-0.8	6.0	6.1	0.1
Lung	9.0	8.4	-0.6	14.2	15.5	1.2
Melanoma	76.7	78.9	2.2	92.4	91.1	-1.3
Breast	78.4	76.1	-2.3	89.2	87.9	-1.3
Cervix	57.6	60.0	2.4	73.7	75.3	1.6
Uterus	77.9	73.4	-4.4	82.2	81.5	-0.7
Ovary	51.0	48.0	-3.0	41.8	42.4	0.6
Prostate	81.0	81.1	0.2	89.7	87.0	-2.7
Testis	94.1	91.7	-2.5	96.2	96.8	0.6
Kidney	65.7	68.1	2.4	68.5	70.2	1.7
Bladder	52.8	61.4	8.5	63.9	67.8	3.9
Brain	41.8	43.8	2.1	20.9	21.8	0.9
Thyroid	84.4	87.2	2.8	95.1	95.3	0.2
Hodgkin lymphoma	100.4	100.0	-0.4	83.7	85.4	1.7
Non-Hodgkin lymphoma	55.4	58.4	3.0	65.5	68.0	2.6
Leukaemia	52.0	62.9	10.9	53.2	64.3	11.1
Unknown primary	14.4	16.0	1.6	17.0	20.9	3.9
Others	40.1	45.2	5.1	48.9	49.7	0.8
Allcancers[3]	45.8	47.0	1.2	64.6	65.6	1.0

1. not adjusted for age.
2. cause-specific minus relative.
3. not adjusted for site.
