# Supplementary material for: Cancer survival for Aboriginal and Torres Strait Islander Australians: a national study of survival rates and excess mortality
Source: Popul Health Metr. 2014 Jan 31;12:1. doi: 10.1186/1478-7954-12-1 (PMC3909914; doi:10.1186/1478-7954-12-1)
Supplement: Additional file 3: Table S4a — Regression analysis of cause-specific mortality for all cancers combined, Australia (excluding Victoria) 1991-2005 (full model). Description: Table S4 including hazard ratios for specific cancer sites. [file 1478-7954-12-1-S3.pdf]

Table 4a Regression analysis of cause-specific mortality for all cancers combined, Australia (excluding Victoria) 1991-2005 (full model).

		HR[1]	(95%CI)
Indigenous[2]			
	1st year after diagnosis	1.50	(1.41-1.60)
	2nd year after diagnosis	1.23	(1.11-1.35)
	3rd year after diagnosis	1.21	(1.07-1.38)
	4th year after diagnosis	1.16	(0.98-1.37)
	5th year after diagnosis	0.99	(0.79-1.23)
Sex			
	Female	0.93	(0.92-0.93)
Age at diagnosis (per year of age)			
	Non-Indigenous	1.03	(1.03-1.03)
	Indigenous	1.02	(1.02-1.02)
Remoteness (per ARIA category)			
	Non-Indigenous	1.05	(1.05-1.06)
	Indigenous	1.13	(1.11-1.16)
Cancer site/type[3]			
	Head & neck	0.73	(0.72-0.75)
	Stomach	2.88	(2.83-2.94)
	Anus	0.97	(0.90-1.03)
	Liver	4.82	(4.69-4.96)
	Pancreas	6.48	(6.37-6.60)
	Lung	4.16	(4.11-4.21)
	Melanoma	0.23	(0.23-0.24)
	Breast	0.37	(0.37-0.38)
	Cervix	0.92	(0.89-0.96)
	Uterus	0.47	(0.45-0.49)
	Ovary	2.15	(2.10-2.21)
	Prostate	0.31	(0.30-0.32)
	Testis	0.15	(0.13-0.17)
	Kidney	0.95	(0.92-0.97)
	Bladder	0.72	(0.70-0.73)
	Brain	5.60	(5.48-5.72)
	Thyroid	0.20	(0.19-0.22)
	Hodgkin lymphoma	0.66	(0.61-0.72)
	Non-Hodgkin lymphoma	1.12	(1.10-1.14)
	Leukaemia	1.24	(1.21-1.27)
	Unknown primary	5.52	(5.44-5.61)
	Others	1.60	(1.57-1.62)

1. Hazard ratio
2. Applies to the reference categories of the interaction terms (i.e. people of median age 59 years and resident in major cities).
3. compared to colorectal cancer.
