# Supplementary material for: Cancer survival for Aboriginal and Torres Strait Islander Australians: a national study of survival rates and excess mortality
Source: Popul Health Metr. 2014 Jan 31;12:1. doi: 10.1186/1478-7954-12-1 (PMC3909914; doi:10.1186/1478-7954-12-1)
Supplement: Additional file 4: Table S5a — Time trends: regression analysis of cause-specific mortality in two years after diagnosis for all cancers combined, Australia (excluding Victoria) 1991-2005 (full model). Description: Table S5 including hazard ratios for specific cancer sites. [file 1478-7954-12-1-S4.pdf]

Table 5a Time trends: regression analysis of cause-specific mortality in two years after diagnosis for all-cancers combined, Australia (excluding Victoria) 1991-2005 (full model).

		HR[1]	(95%CI)
Indigenous[2]		1.54	(1.42-1.66)
Sex			
	Female	0.95	(0.94-0.95)
Age at diagnosis (per year of age)			
	Non-Indigenous	1.03	(1.03-1.03)
	Indigenous	1.02	(1.02-1.02)
Remoteness (per ARIA category)			
	Non-Indigenous	1.06	(1.06-1.07)
	Indigenous	1.16	(1.13-1.19)
Year of diagnosis (per year)			
	Non-Indigenous	0.98	(0.98-0.98)
	Indigenous	0.99	(0.98-1.00)
Cancer site/type[3]			
	Head & neck	0.76	(0.75-0.78)
	Stomach	3.09	(3.03-3.15)
	Anus	0.89	(0.82-0.97)
	Liver	5.30	(5.14-5.46)
	Pancreas	6.85	(6.72-6.98)
	Lung	4.40	(4.34-4.46)
	Melanoma	0.20	(0.19-0.20)
	Breast	0.26	(0.25-0.26)
	Cervix	0.92	(0.87-0.96)
	Uterus	0.46	(0.44-0.49)
	Ovary	1.97	(1.91-2.03)
	Prostate	0.24	(0.23-0.25)
	Testis	0.19	(0.16-0.22)
	Kidney	1.06	(1.03-1.09)
	Bladder	0.74	(0.72-0.77)
	Brain	6.15	(6.01-6.29)
	Thyroid	0.24	(0.22-0.26)
	Hodgkin lymphoma	0.70	(0.64-0.77)
	Non-Hodgkin lymphoma	1.19	(1.17-1.22)
	Leukaemia	1.35	(1.32-1.39)
	Unknown primary	6.29	(6.19-6.40)
	Others	1.69	(1.66-1.72)

1. Hazard ratio
2. Applies to the reference categories of the interaction terms (i.e. people of median age 59 years in 1998).
3. compared to colorectal cancer.
