# Supplementary material for: Cancer survival for Aboriginal and Torres Strait Islander Australians: a national study of survival rates and excess mortality
Source: Popul Health Metr. 2014 Jan 31;12:1. doi: 10.1186/1478-7954-12-1 (PMC3909914; doi:10.1186/1478-7954-12-1)
Supplement: Additional file 5: Table S8 — Regression analysis for all cancers combined including an interaction term for year of diagnosis by remoteness category, hazard ratio (95% confidence interval), Australia (excluding Victoria) 1991-2005. Description: Separate regression analyses for Indigenous and non-Indigenous cases including interaction term to assess whether time trends vary by remoteness of residence. [file 1478-7954-12-1-S5.pdf]

Table 8 Regression analysis[1] for all-cancers combined including an interaction term for year of diagnosis by remoteness category, hazard ratio (95% confidence interval), Australia (excluding Victoria) 1991-2005.

	Non-Indigenous		Indigenous	
Sex	0.95	(0.94-0.95)	0.95	(0.88-1.03)
Age	1.03	(1.03-1.03)	1.02	(1.02-1.02)
Year	0.98	(0.98-0.98)	1.01	(0.99-1.03)
Remoteness (per ARIA category)	1.06	(1.05-1.07)	1.11	(1.06-1.16)
Year by remoteness interaction	1.00	(1.00-1.00)	0.99	(0.99-1.00)
Site[2]				
Head & neck	0.75	(0.73-0.77)	1.52	(1.26-1.84)
Stomach	3.09	(3.03-3.16)	2.67	(2.11-3.37)
Anus	0.88	(0.81-0.96)	1.11	(0.62-2.00)
Liver	5.34	(5.19-5.50)	3.56	(2.84-4.48)
Pancreas	6.87	(6.74-7.00)	5.30	(4.29-6.55)
Lung	4.41	(4.35-4.47)	3.49	(2.97-4.10)
Melanoma	0.20	(0.19-0.20)	0.20	(0.10-0.39)
Breast	0.26	(0.25-0.26)	0.32	(0.25-0.41)
Cervix	0.91	(0.86-0.95)	1.04	(0.81-1.34)
Uterus	0.46	(0.44-0.49)	0.46	(0.32-0.66)
Ovary	1.98	(1.92-2.04)	1.21	(0.86-1.71)
Prostate	0.24	(0.23-0.25)	0.31	(0.22-0.42)
Testis	0.19	(0.16-0.22)	0.43	(0.17-1.04)
Kidney	1.06	(1.03-1.09)	0.87	(0.61-1.24)
Bladder	0.74	(0.72-0.76)	0.94	(0.67-1.31)
Brain	6.19	(6.04-6.33)	3.06	(2.26-4.14)
Thyroid	0.24	(0.22-0.26)	0.28	(0.16-0.48)
Hodgkin lymphoma	0.71	(0.65-0.78)	0.00	-
Non-Hodgkin lymphoma	1.19	(1.16-1.22)	1.62	(1.25-2.10)
Leukaemia	1.35	(1.32-1.38)	1.72	(1.33-2.23)
Unknown primary	6.32	(6.22-6.42)	4.43	(3.70-5.31)
Others	1.69	(1.66-1.72)	1.59	(1.32-1.90)

1. cause-specific mortality in two years after diagnosis, stratified by Indigenous status
2. compared to colorectal cancer.
